# Contact with grandparents and young people’s explicit and implicit attitudes toward older adults

**DOI:** 10.1186/s40359-023-01344-7

**Published:** 2023-09-26

**Authors:** Tiansi Liao, Cuo Zhuoga, Xiaochen Chen

**Affiliations:** 1 Department of Psychology, Renmin University of China, Beijing, 100872 China; 2 Laboratory of Department of Psychology, Renmin University of China, Beijing, China; 3 Interdisciplinary Platform of Philosophy and Cognitive Science, Renmin University of China, Beijing, China

**Keywords:** Intergenerational contact, Grandparent–grandchild relationship, Explicit and implicit attitudes, Ageism, Young people

## Abstract

**Background:**

Given the dramatic rise in population aging and widespread negative attitudes toward older people, it is necessary to understand the factors that affect age-related attitudes among young people in order to improve intergenerational solidarity and reduce ageism. The current study examined young people’s contact with their grandparents and attitudes toward older people on both explicit and implicit levels.

**Method:**

The sample included 146 Chinese college students (*M*_*age*_ = 21.50 yrs, *SD* = 2.23, 101 females). Participants completed a questionnaire concerning contact with their grandparents(contact quantity and contact quality), perceived typicality of their grandparents, intergroup anxiety, inclusion of other in the self, and explicit attitudes toward older people (aged 65 years or older) in general. Participants were also invited to complete a single-category implicit association test (SC-IAT) to assess their implicit attitudes toward older people.

**Results:**

The findings indicated that both quantity and quality of contact with grandparents predicted better explicit attitudes toward older people, and contact effects were stronger when one’s grandparents were perceived as being typical of older adults. Contact quantity (not quality) was associated with more favorable implicit attitudes only when one’s grandparents were perceived as highly typical older adults. Contact effects on explicit attitudes were mediated by intergroup anxiety and inclusion of other in the self.

**Conclusion:**

Our findings on the positive effects of contact with grandparents underscore the importance of promoting intergenerational contact within the family as a starting point to reduce prejudice toward older adults in age-segregated modern societies. Current results also provide insights on how to extend the benefits of grandparent-grandchild contact outside the family by promoting the perceived typicality of one’s grandparents.

**Supplementary Information:**

The online version contains supplementary material available at 10.1186/s40359-023-01344-7.

## Background

Globally, the older population is rapidly growing. The current global population of those aged 65 and above is approximately 750 million, and projections suggest that the number will reach 1.5 billion by 2050. At that point, about one in every six people in the world will be aged 65 years or above [1].

While rising life expectancy is a positive outcome, the expanding older population is likely to encounter challenges, including more negative attitudes toward older adults [2]. Negative attitudes toward older adults are prevalent worldwide, even in Eastern cultures that traditionally value respect for older people [2–4]. During the COVID-19 pandemic, the vulnerability narratives of older people (e.g., higher infection and mortality rates) in media exacerbated the already widely embedded negative impressions of older adults as feeble, dependent, and a societal burden [5]. The high prevalence of ageism (stereotyping, prejudice, and discrimination against older adults) is a significant problem because ageism has deleterious effects on the mental and physical health of older adults, such as a reduction in self-esteem [6], a decrease in cognitive tasks [7], an increased risk of cardiovascular diseases, and even a shortened lifespan [8].

Given the dramatic rise in population aging and widespread negative attitudes toward older people, it is necessary to understand the factors that affect age-related attitudes among young people to improve intergenerational solidarity and reduce ageism. Thus, using contact theory as a guiding framework [9, 10], we examined the relationship between grandparent and grandchild (GP–GC) contact and young individuals’ explicit and implicit attitudes toward older people. We also explored the possible moderating and mediating mechanisms underlying contact effects. The current study was performed with a sample of Chinese college students from mainland China, where multigenerational cohabitation is very common [11] and where almost a quarter of all those aged over 65 in the world currently live.

### Explicit and implicit ageism

College students, in both Western and Eastern cultural contexts, generally hold negative perceptions about aging and older adults, associating old age with sickness, frailty, loneliness, boring and grumpy [12–14]. However, there is also evidence showing that college students have neutral or positive attitudes toward older people [15, 16]. Inconsistencies in findings could be partly attributed to distinct measures of ageism.

On the basis of the dual-attitude model, explicit and implicit attitudes toward the same object may coexist in memory [17]. Explicit attitudes are controllable and conscious, and thus susceptible to self-presentational and social-evaluative concerns. Implicit attitudes rely on automatic activation to operate out of awareness and are thus less susceptible to strategic control.

In the domain of ageism, people’s self-reported explicit attitudes have been found to be associated with social desirability [18], and there lacks an evident association between explicit and implicit measures of age-related attitudes. For example, in a study on age bias across a person’s lifespan, Chopik and Giasson found that, although explicit preference for younger individuals was lowest among older people, implicit preference for the young was highest among older people [19]. In another study examining Chinese undergraduates’ explicit and implicit age stereotypes, Zuo et al. found that the implicit stereotypes of older adults were more negative than those of younger people, whereas explicit measures did not reveal any age bias [14]. These data highlight the importance of including both explicit and implicit measures when studying attitudes toward older people.

### Grandparent–grandchild (GP–GC) intergenerational contact and attitudes toward older people

Intergenerational contact is an important factor that potentially impacts a young person’s views on older people. According to the intergroup contact theory [9], intergroup contact, under the right circumstances (e.g., noncompetitive or institutionally supported), could reduce prejudice and improve intergroup attitudes. Empirical studies have shown that positive intergenerational contact improves young individuals’ attitudes toward older people [20].

The GP–GC relationship in family contexts provides young people with special opportunities for intimate contact with older adults in today’s age-segregated communities. In Pettigrew’s reformulation of contact theory [9, 10], the author pointed out that intergroup relationships featuring extensive and repeated contact may be particularly crucial for attitude change. In an enduring, intimate family relationship context, GP–GC contact is typically more satisfying than intergenerational contact outside of the family [21]. Hence, it is a powerful form of intergenerational contact. Previous literature on intergroup contact in family contexts from Western countries revealed that high-quality contact with grandparents was associated with more favorable feelings toward older people among adolescents and young adults [21, 22]. In China, multigenerational cohabitation and grandparents’ help in raising grandchildren are common [11]. Indeed, most contact between Chinese adolescents or young adults, and older people occurs in the family [3]. However, studies examining GP–GC contact and attitudes toward older people in China are rare.

A traditional Confucian norm *xiao* (i.e., filial piety), which emphasizes respect for and obedience to elder family members, is still upheld in modern China [23]. On the one hand, such an age-related social norm might facilitate the generalization of GP-GC contact effects to all older adults [9]. On the other hand, as a virtue prescribing family roles, filial piety might lead to subtyping of one’s grandparents (e.g., “my grandparents are exceptions of older people”), which in turn could prevent positive GP-GC contact from being generalized to non-family members [24, 25]. Our study could shed light on possible cultural similarities and differences in intergenerational contact effects.

One unsolved issue in the intergenerational contact literature concerns the function of contact quantity versus contact quality. Some researchers found that more frequent contact with older people was related to more accurate knowledge about aging and more positive attitudes toward older people [25, 26]. Other scholars maintained that quality, but not the quantity of intergenerational contact, was related to ageism [20, 27]. Another gap in the intergenerational contact literature concerns the measurement of age-related attitudes. Most studies have relied on self-report scales to assess people’s explicit attitudes toward older adults. Only a few intergroup contact studies have included both explicit and implicit attitude measures, and the findings are mixed. Some studies revealed that implicit outgroup attitudes depend on the quantity of contact [28, 29]. In contrast, other studies found that contact effects on implicit attitudes were driven by the quality of contact [30]. Thus, the specific linkages between particular aspects of contact (quantity vs. quality) and different dimensions of attitudes remain inconclusive.

### Moderating and mediating processes

How to facilitate the generalization of contact effects from one’s own grandparents to all older adults outside the family context is a prominent issue in reducing ageism. Previous studies on intercultural contact have suggested that the extent to which encountered outgroup members are perceived to be typical of their group can play a key role in moderating the effects of positive intergroup contact on generalized attitudes. Specifically, the more the particular outgroup members encountered are perceived as typical representatives of their group, the stronger the association between contact and favorable attitudes toward the outgroup as a whole [31, 32]. In GP–GC contact, it is reasonable to expect that contact effects are stronger when one’s grandparents are perceived as typical representatives of older adults.

The underlying mechanisms of contact effects have also drawn wide attention from researchers. Intergroup anxiety has been demonstrated as an effective mediator of intergroup contact [33]. Intergroup anxiety is defined as the feelings of awkwardness and apprehension when envisioning or being in a contact situation with outgroup members, which may be attributed to expected misunderstanding, embarrassment, or rejection [34]. Positive contact experiences are able to reduce anxiety, leading to a decrease in prejudice. Evidence for the mediating role of intergroup anxiety comes from studies on different types of intergroup contact, including intergenerational contact [25, 31].

Another candidate mediating variable is inclusion of other in the self (IOS). IOS refers to an overlap between close others and the concept of the self, which is a defining characteristic of close relationships [35]. Previous studies on close relationships have empirically identified a self–other overlap with spouses and close friends [35, 36]. Self-expansion theory [37, 38] further proposes that if the individual characteristics (e.g., personality traits) of close others become automatically associated with the self, then collective characteristics (e.g., group membership) might also be associated with the self. If the outgroup comes to be included in the self, then outgroup members would obtain the advantages of ingroup members, such as feeling empathy for their troubles, taking pride in their successes, and seeing them in a positive light. Studies on inter-ethnic friendships have shown that cross-group friendships lead to more IOS, which, in turn, is related to improved intergroup attitudes [36, 38, 39]. Following the same logic, we suspect that in a GP–GC relationship, young people might include their grandparents in the self, which serves as a mediator between GP–GC contact and improved attitudes toward older people.

### The current study

In the current study, we examined young people’s contact with grandparents and their attitudes toward older adults. We assessed contact quantity as well as contact quality and included both explicit and implicit measures of age-related attitudes. Hence, the specific linkage between different aspects of GP–GC contact and different dimensions of attitudes could be explored. Given the limited and mixed findings in previous research, our analysis of whether quantity and quality of contact are differentially linked with explicit and implicit attitudes toward older people is exploratory. We also examined the possible moderating and mediating mechanisms underlying GP–GC contact, as well as a combined model of moderated mediation. Specifically, we hypothesized that contact effects would be stronger when one’s grandparent was perceived as a highly typical older adult. As to the mediating processes, we aimed to replicate a powerful intergroup contact mediator (i.e., intergroup anxiety) and to test a new mediator (i.e., IOS) in the context of GP-GC contact and explicit attitudes toward older people. Given the automatic nature of implicit attitudes [17], we expect that the conscious mediating processes are not involved in the contact-implicit attitudes linkage. We conducted this study with a sample of Chinese college students because population aging is a prominent social issue in China [40] and because Chinese young people are understudied in the intergenerational contact literature.

## Method

### Participants and Procedure

A total of 162 college students were initially recruited from a large public university in northeastern China. Participants completed a questionnaire, which had two counterbalanced sections. Section A included questions concerning relationships with their four biological grandparents. Section B measured explicit attitudes toward older people (aged 65 years or older) in general and toward one’s own aging. Participants reported on deceased grandparents if they could clearly recall the relationship. To assess implicit attitudes toward older adults, participants were also invited to complete a single-category implicit association test (SC-IAT). The order of explicit (i.e., questionnaire) and implicit attitude measures (i.e., SC-IAT) was counterbalanced between participants. The analyses revealed no order effect. Sixteen participants were excluded from the final analysis due to excessive missing data on the questionnaire (*n* = 7) or extreme response times on the SC-IAT (*n* = 9). The final analytic sample comprised 146 college students (*M*_*age*_ = 21.50 yrs, *SD* = 2.23, 101 females).

All participants voluntarily and anonymously participated in the study and were informed that they could stop at any time. They were subsequently debriefed and received an honorarium of 15 RMB for their participation.

### Measures

#### Contact measures

Two items measured the frequency of contact (i.e., contact quantity) with each grandparent. The items were adapted from Zhang et al.’s study (e.g., “How often do you talk to and engage in an informal conversation with this grandparent?”; 1 = *not frequently at all* – 7 = *very frequently*) [25]. The alpha coefficients were 0.84, 0.86, 0.80, and 0.83, for maternal grandfather, maternal grandmother, paternal grandfather, and paternal grandmother, respectively.

Two questions evaluated the quality of contact in the GP-GC relationships. Participants were asked how well they “get along with” the grandparent (*very poorly—very well*), and how “emotionally close” they felt to the grandparent (*very distant—very close*). The scores of both items ranged from 1 to 5, with higher scores indicating better contact quality. These questions were reliable across grandparent relationships (with Cronbach’s αs ranging from 0.87 to 0.91).

#### Perceived typicality

Perceived typicality of each grandparent was assessed using a single item (“Is your paternal grandfather/paternal grandmother/maternal grandfather/maternal grandmother typical of all older adults in general?”). The range of the 7-point response scale was 1 (*not at all*) to 7 (*very typical*), with higher scores indicating more perceived typicality. A similar measure has been employed in previous research [31].

#### Inclusion of other in the self (IOS)

Inclusion of one’s grandparents in the self was determined based on a pictorial item [41]. Seven pairs of overlapping circles were demonstrated; participants were asked to indicate the pair that best reflected the nature of their GP–GC relationship. Higher scores reveal greater inclusion of grandparents in the self. Relationships with each grandparent were assessed separately.

#### Intergroup anxiety

Intergroup anxiety was assessed using the Intergroup Anxiety Scale developed by Stephan and Stephan [34]. The scale consists of 10 adjectives indicating the feelings of interactions with out-group members, including seven negatively valenced adjectives (e.g., irritated and awkward) and three positively/reversed-scored adjectives (i.e., confident, accepted, and happy). Participants were asked to rate their corresponding feelings from 1 (*not at all*) to 7 (*very much*), based on their experience of interactions with complete strangers who were 65 or older. Responses were scored in a way that higher scores indicate greater intergroup anxiety (α = 0.88).

#### Attitudes toward older people

Explicit and implicit attitudes toward older people were assessed separately.

Explicit attitudes toward older people were evaluated using a Chinese version of Kogan’s Attitude toward Older People Scale (KAOP) [42]. This KAOP comprises 25 items related to older adults. Fourteen items are negatively worded (e.g., “The elderly are irritable, grouchy and unpleasant.”), whereas the remaining items are positively worded (e.g., “The elderly grow wiser with advancing age.”). The scale is designed as a summed Likert attitude scale with 7-point response categories, ranging from 1 (*strongly disagree*) to 7 (*strongly agree*). Scores on the negatively worded items need to be reverse-coded to obtain the total score. Higher total scores represent more positive attitudes toward older people (α = 0.83).

Implicit attitudes toward older people were evaluated using the SC-IAT procedure. The SC-IAT is a modification of the Implicit Association Test that assesses the strength of an evaluative association with a single attitude object [43]. The current SC-IAT contained two stages, all of which participants completed in the same way. As shown in Table 1, each stage contained 24 practice trials, followed by 72 test trials. In the 1st stage (Blocks 1 and 2), target words representing older adults and positive adjectives were classified as the *F* key, and negative adjectives were classified as the *J* key. To preempt response bias, target words representing older adults, positive words, and negative words were shown in a 1:1:2 ratio so that the corrected responses were distributed equally between the *F* and *J* keys. In the 2nd stage (Blocks 3 and 4), positive adjectives were classified as the *F* key, and negative adjectives and target words representing older adults were classified as the *J* key. Words representing older adults, positive words, and negative words were shown in a 1:2:1 ratio so that the corrected responses were distributed equally between the *F* and *J* keys.

**Table 1:** SC-IAT Procedure

Block	Trials	Function	Left-key (“*F*”) response	Right-key (“*J*”) response
1	24	Practice	Positive words + older adults	Negative words
2	72	Test	Positive words + older adults	Negative words
3	24	Practice	Positive words	Negative words + older adults
4	72	Test	Positive words	Negative words + older adults

Attributive words and target words used in the SC-IAT were drawn from a pilot study examining Chinese college students’ perceptions of older people. One hundred and seventy college students recruited online were asked to answer an open-ended image-of-aging question [44]: “Given the five words that first come to your mind when you think of persons 65 years and older.” Responses were given a quantitative assessment by asking three external evaluators (two graduate students majoring in Chinese and one graduate student majoring in applied psychology) to assign a score on a Likert-type scale ranging from − 5 (*extremely negative*) to + 5 (*extremely positive*) to each word in reference to an older adult. The reliability of raters was high, ICC = 0.83, *p* < .01. Eight frequently mentioned nouns with neutral meanings (scores ranging from − 2 to + 2) were chosen as target words to represent older adults, such as *retirement* and *walking stick*. The attribute words included 16 frequently mentioned adjectives. Half were positive words for the positive evaluative dimension (e.g., *kind* and *wise*), whereas the other half were negative words for the negative dimension (e.g., *feeble* and *old-fashioned*).

According to the SC-IAT procedures established by Karpinski and Steinman [43], each stage was preceded by a series of instructions focusing on the dimensions of the categorization task and suitable key responses. Each target word or attributive word was centered on the computer screen. Category reminder labels were positioned at the bottom fourth of the screen. The stimulus word stayed on the screen for 1,500ms or until the participants responded. If the participants did not respond within 1,500 ms, a reminder to “Please respond more quickly!” appeared for 500 ms. After each response, the participants were given feedback about the accuracy of their responses. A red X in the center of the screen for 150 ms followed incorrect responses; a green √ in the center of the screen for 150 ms followed each correct response.

Only test blocks (Blocks 2 and 4) were used to calculate the *D*-values. Responses of less than 350 ms were removed, and error responses were substituted with the block mean and an error penalty of 400 ms. The mean response times of Block 2 were subtracted from the mean response times of Block 4. The standard deviation of all correct response times within Blocks 2 and 4 was used to divide this quantity. Hence, the *D*-scores represent more positive than negative associations with older adults. In other words, higher *D*-scores indicate better implicit attitudes toward older people. A reliability analysis following the procedures outlined by Karpinski and Steinman revealed a reasonable level of internal consistency of the SC-IAT measures (adjusted *r* = .73) [43].

#### Covariates

Gender, age, and aging anxiety were also included as control variables in the main analyses. Previous literature suggested possible gender differences in attitudes toward older people, although empirical findings on this issue are not conclusive [14, 45]. Aging anxiety refers to concerns related to the negative aspects of one’s personal aging, such as loss of one’s independence, social relationships, physical and mental health, and ultimately, one’s very existence [46]. Aging anxiety has been found to be positively linked with ageist attitudes [47, 48], since older adults may present a threat to young people by reminding them of the inevitable consequences of their own aging [49]. In the current study, aging anxiety was measured using four items asking participants how they felt about personal aging [20]. An example item is “I am concerned that my mental abilities will suffer when I am old.” The 5-point response scale ranged from 1 (*strongly disagree*) to 5 (*strongly agree*). The higher the scores, the greater the aging anxiety (α = 0.75).

## Results

### Analytic plan

The results are presented in four main sections. First, descriptive and correlation analyses among key variables were performed to obtain preliminary evidence for our hypotheses regarding the distinctive linkages between particular aspects of GP–GC contact (quantity vs. quality) and explicit and implicit attitudes toward older people. Next, linear regression analyses were carried out to further assess the contact effects and possible moderation of typicality. We then tested intergroup anxiety and IOS as mediating variables. Finally, more complex moderated mediation models were employed to determine whether the mediating effects were moderated by typicality.

In all analyses, gender (0 = female), age, and aging anxiety were treated as control variables. Analyses without covariates showed essentially the same pattern of results. Since people rely on their more active relationships when developing impressions of an out-group as a whole [21], the current analyses focused on the relationship that has the most frequent contact. Two participants were excluded from the analyses concerning contact quality as they did not report contact with at least one grandparent of each lineage.

### Descriptive analyses

As demonstrated in Table 2, explicit and implicit attitudes toward older adults were not significantly correlated (*r* = − .09, *n.s.*). Contact with grandparents was positively correlated with explicit attitudes (*rs* = 0.36 and 0.30 for contact quantity and contact quality respectively, *ps* < 0.001), whereas the correlations between intergenerational contact and implicit attitudes were not significant. Contact measures were also positively correlated with IOS (*rs* = 0.55 and 0.67 for contact quantity and contact quality respectively, *ps* < 0.001) and negatively correlated with intergroup anxiety (*rs* = − 0.19 for both contact quantity and contact quality, *ps* < 0.05). In addition, the two potential mediators were significantly correlated with explicit attitudes in the expected direction but were unrelated to implicit attitudes.

**Table 2:** Summary of intercorrelations, means, and standard deviations of key variables

	1	2	3	4	5	6	7
1. Contact quantity	1.00						
2. Contact quality	0.57^***^	1.00					
3. IOS	0.55^***^	0.67^***^	1.00				
4. Intergroup anxiety	− 0.19^*^	− 0.19^*^	− 0.10	1.00			
5. Typicality	0.32^***^	0.55^***^	0.36^***^	0.08	1.00		
6. Explicit attitudes	0.36^***^	0.30^***^	0.34^***^	− 0.48^***^	0.03	1.00	
7. Implicit attitudes	− 0.09	− 0.10	0.00	− 0.04	− 0.22^**^	− 0.09	1.00
*M*	4.48	4.12	4.39	3.36	4.85	111.92	− 0.12
*SD*	1.50	1.07	1.72	0.85	1.57	15.03	0.32

*Note*. IOS = Inclusion of Other in the Self. ^*^p < .05, ^**^p < .01, ^***^p < .001

### Contact Effects and Moderation analyses

To explore contact effects on attitudes toward older people and the possible moderating role of perceived typicality of one’s grandparent, attitude scores were regressed on contact measure, typicality, and contact by typicality interaction terms. We first examined contact effects regarding the quantity of contact, followed by analyses of the quality of contact. For each contact measure, the regression model was performed twice: once for explicit attitudes and once for implicit attitudes.

The data (see Table 3) indicated that both contact measures significantly predicted explicit attitudes toward older adults (*B*_*contact quantity*_*=* 4.16, *B*_*contact quality*_ = 9.73, *ps* < 0.001), such that more GP–GC contact and contact of higher quality were related to better explicit attitudes toward older people. The interaction term between contact quality and typicality in predicting explicit attitudes was also significant (*B* = 0.96, *p* < .01). The decomposition of this moderating effect [50] indicated that contact quality had a stronger effect under high (*B* = 11.24, *p* < .001) than under low (*B* = 8.21, *p* < .001) typicality. Neither contact measure significantly predicted implicit attitudes toward older adults. A significant contact quantity by typicality interaction (*B* = 0.05, *p* < .01) indicated that more GP-GC contact was associated with improved implicit attitudes only when typicality was high (*B*_*high typicality*_ = 0.07, *p* < .01; *B*_*low typicality*_ = − 0.01, *n.s.*).^1^

**Table 3:** Summary of results for moderation analyses (top panel: contact quantity; bottom panel: contact quality)

Predicator	Explicit attitudes				Implicit attitudes		
	*B*	*SE*	*t*		*B*	*SE*	*t*
Contact quantity	4.16	0.86	4.86^***^	0.00		0.02	− 0.07
Typicality	− 0.97	0.94	-1.04	− 0.03		0.02	-1.28
Quantity×typicality	− 0.13	0.39	− 0.34	0.05		0.01	3.94^***^
*R*^2^	0.15			0.14			
Predicator	Explicit attitudes				Implicit attitudes		
	*B*	*SE*	*t*		*B*	*SE*	*t*
Contact quality	9.73	1.75	5.55^***^	0.03		0.04	0.77
Typicality	-1.04	0.94	-1.11	− 0.04		0.02	-1.64
Quality×typicality	0.96	0.30	3.26^**^	0.05		0.03	1.66
*R*^2^	0.19			0.06			

Note.^**^p < .01, ^***^p < .001

### Mediation analyses

Given that significant contact effects were found only on explicit measures of age-related attitudes, mediation analyses focused only on contact–explicit attitudes relationships. The protocols reported by Preacher and Hayes [51] were used to determine whether intergroup anxiety and/or IOS can mediate the positive associations between intergenerational contact (contact quantity and contact quality) and young individuals’ attitudes toward older people. To this end, we used the PROCESS macro for SPSS [52], by which bootstrapping techniques were employed to estimate the direct and total effects of a predictor variable on an outcome variable, and the indirect effects through one or more mediators. These analyses have the advantage of greater statistical power without assuming multivariate normality in the sampling distribution. If the bias-corrected 95% confidence interval (*BC CI*) does not include zero, an indirect effect (*IE*) is considered significant.^2^

In the mediation analysis of the contact quantity–attitude relationship, the significant total and direct effects of contact quantity on attitudes were observed (*B =* 3.92, *p <* .001, and *B =* 1.82, *p* < .05, respectively). The significant total indirect effects through intergroup anxiety and IOS were also observed, *IE =* 2.10, *BC CI* [0.93, 3.57], as well as the specific indirect effects through intergroup anxiety, *IE =* 0.98, *BC CI* [0.23, 1.90], and IOS, *IE =* 1.13, *BC CI* [0.26, 2.20]. This indicates that both intergroup anxiety and IOS can mediate the relationship between contact quantity and young individuals’ attitudes toward older people.

Similarly, in the mediation analysis of the contact quality–attitude relationship, the significant total and direct effects of contact quality on attitudes were observed (*B =* 9.13, *p <* .001 and *B =* 3.65, *p* < .05, respectively). The significant total indirect effect through intergroup anxiety and IOS was observed, *IE =* 5.48, *BC CI* [ 2.67, 8.64], as well as the specific indirect effects through intergroup anxiety, *IE =* 2.87, *BC CI* [1.02, 5.21], and IOS, *IE =* 2.61, *BC CI* [0.64, 5.00]. This indicates that intergroup anxiety and IOS also mediate the relationship between contact quality and young people’s age-related attitudes.

### Moderated mediation

Next, analyses were conducted to examine whether the above-documented mediations were moderated by the perceived typicality of the most frequently contacted grandparent. First, the protocols reported by Muller et al. were used to examine moderated mediation [53]. Thus, two criteria had to be met. First, the main effect of the independent variable on the dependent variable had to be significant. Second, either the interaction between the moderator and the independent variable had to significantly predict the mediator and the mediator significantly predict the dependent variable while controlling for the interactions between the moderator and the independent variable, or the interaction between the mediator and the moderator had to significantly predict the dependent variable and the independent variable significantly predict the mediator. In addition, the conditional indirect effect approach of Preacher et al. was employed to determine the indirect effects and corresponding 95% CIs [54].

Separate analyses were performed for different contact measures (contact quantity and contact quality) and each mediator (intergroup anxiety and IOS). Four sets of moderated mediation analyses were conducted in total. Only the mediating role of intergroup anxiety in the linkage between contact quality and explicit attitudes was moderated by typicality. The results of these analyses are reported in detail below:

The interaction between contact quality and typicality in predicting intergroup anxiety was significant (*B* = − 0.04, *p* < .05; Model 1 in Table 4). The decomposition of this interaction revealed that contact quality exhibited a stronger effect on intergroup anxiety under high (*B* = − 0.50, *p* < .01) than under low (*B* = − 0.37, *p* < .01) typicality. In addition, intergroup anxiety significantly predicted explicit attitudes toward older adults (*B* = -6.63, *p* < .001) while controlling for contact quality, typicality, and the interaction term (see Model 2 in Table 4). Therefore, the criteria for documenting moderated mediation were satisfied. The results of the bootstrapping approach [54] indicated that the mediation effect of intergroup anxiety was stronger when typicality was higher, high typicality (*M* + 1*SD*): *IE =* 3.84, *BC CI* [0.88, 7.69]; typicality at mean level: *IE =* 2.87, *BC CI* [1.02, 5.21]; low typicality (*M* − 1*SD*): *IE =* 2.09, *BC CI* [0.54, 4.27]. 3

**Table 4:** Assessment of the moderated mediation

Predictor	Model 1			Model 2		
	Outcome: Intergroup anxiety			Outcome: Explicit attitudes		
	*B*	*SE*	*t*	*B*	*SE*	*t*
Contact quality (*X*)	− 0.44	0.10	− 4.26^***^	6.75	1.71	3.94^***^
Intergroup anxiety (*M*_*e*_)				− 6.63	1.38	− 4.80^***^
Typicality (*M*_*o*_)	0.10	0.06	1.74	− 0.48	0.89	− 0.54
*X* × *M*_*e*_	− 0.04	0.02	− 2.45^*^	0.66	0.28	2.38^*^
*M*_*e*_ ×*M*_*o*_				− 0.63	0.95	− 0.66
	*R*^2^= 0.13			*R*^*2*^ = 0.32		

Note.^*^p < .05, ^***^p < .001

## Discussion

Rapid population aging occurs against a backdrop of increasing research, revealing that ageism is the most frequent type of prejudice [55]. Therefore, it is important to identify the factors that lead to the development of age-related attitudes in young people, so as to reduce ageism. The present work approached this issue by examining the associations between Chinese young adults’ contact experiences with their most-frequent-contact grandparent and their attitudes toward older people at explicit and implicit levels. Our findings support and extend the intergenerational contact literature in several ways.

First, aligning with previous research [14, 29, 56], the current study demonstrated the disassociation of explicit and implicit attitudes toward older people. There was no significant correlation between self-report attitudes and the SC-IAT scores. More importantly, the current study revealed distinctive linkages between particular aspects of GP–GC contact and different dimensions of attitudes toward older people. Both contact quantity and contact quality with the most frequent GP–GC relationship were positively associated with explicit attitudes toward older people, while only contact quantity under the condition of high typicality was related to implicit attitudes toward older people. Based on the dual-attitude accounts [17, 57], individuals hold a deliberative, explicit attitude, which can be controlled, and a spontaneous, implicit attitude, which is automatic. When an individual experiences intergroup contact—irrespective of how positive or intimate that contact is—that person is also subjective to mere exposure effects. Given the automatic nature of implicit attitudes, it is understandable that contact quantity plays a primary role in implicit attitudes. The current study is one of the few in the broader intergroup contact studies and in the specific realm of intergenerational contact, to include both explicit and implicit attitude measures. Our results underscore the importance of attending to different types of attitudes as outcomes of inter-group contact.

In addition, our study supports and improves the existing models of intergenerational contact by investigating the moderation and mediation mechanisms. Consistent with our hypothesis derived from the group salience literature [31, 32, 58], current results showed that perceived typicality affects the generalization of contact effects. If one’s most frequently contacted grandparent was perceived as being typical of older adults, then contact effects on intergenerational attitudes became stronger. Analyses of moderated mediation also revealed that the linkage between contact quality and reduced intergroup anxiety was stronger when typicality was high vs. low. These findings align with Brown and Hewstone’s propositions [32]. If group membership is readily associated with outgroup members, then positive contact experiences with specific outgroup members can better generalize to the whole outgroup. The lack of moderation in the second path of the mediation (i.e., the link between intergroup anxiety and attitudes) is probably because both intergroup anxiety and explicit attitudes toward older people are group-level variables.

The current study also tested a well-established mediator (i.e., intergroup anxiety) and a relatively new mediator (i.e., IOS) in the context of GP–GC intergenerational contact. In line with previous studies on intergroup contact [33, 59], in the current study, the positive effects of contact quantity and contact quality were partly explained by intergroup anxiety: more contact (and contact of higher quality) with one’s grandparents was linked with reduced anxiety about intergenerational encounters, which, in turn, was related to better explicit attitudes toward older people. Moreover, our results suggest that IOS is another significant mediator underlying GP–GC contact effects. More contact (and contact of higher quality) with a most frequently contacted grandparent were associated with greater overlap between the concept of the self and the close grandparent, which, in turn, was related to better attitudes toward all older adults. To our knowledge, no previous study has explored IOS as an intergroup contact mediator in a GP–GC relationship. Unlike other social categories with more rigid boundaries (e.g., gender and race), age encompasses categories that every living person potentially joins. It is plausible that as young people incorporate their closest grandparent (i.e., an outgroup member in terms of age) into the self, they would be more likely to accept the older age group (i.e., the outgroup) as an ingroup that they will eventually join. It is worth pointing out that, unlike most previous research in intimate cross-group contact [36, 39], the current study measured including the closest grandparent into the self rather than the overlap between the self and the outgroup (i.e., older adults). More direct measures on the inclusion of the outgroup in the self are needed in future GP–GC contact research to test our speculation. In contrast to explicit attitudes, the effects of GP-GC contact on implicit attitudes were unmediated. These findings are in line with previous intergroup contact research including both explicit and implicit attitudes [60, 61]. Given its automatic nature, it makes sense that mediating processes do not play a role in changing implicit attitudes.

Although we believe that this study makes meaningful contributions to the literature on the linkages between intergenerational contact and age-related attitudes, we acknowledge its limitations. One limitation was the issue of directionality. Since the current study is cross-sectional, caution needs to be taken when making causal inferences about the linkages between GP–GC contact and attitudes toward older people. Experimental [36] and longitudinal [31] designs in the wider intergroup contact literature, however, have provided evidence for the causal direction from contact to attitudes. In addition (and may be specific to the present context), the GP–GC relationship is a lifelong association for the young people in the present study, which exists before they developed attitudes toward older people. Nevertheless, longitudinal work is advocated on the GP–GC relationship to better understand the power of GP–GC contact to affect general attitudes toward older people.

Another limitation of our study is its generalizability. We relied on a relatively small college student sample (aged 18–25 years old) to represent young Chinese people. However, previous research has suggested that age, educational level, and work experience are all related to attitudes toward older people [45]. Studies with large representative samples of young people are needed to cross-validate the current findings. Thirdly, it might be interesting for future studies to explore the development of explicit and implicit age-related attitudes and possible age differences in the role of GP–GC contact in shaping ageism. For example, at what point do children start showing ageism? Which age group is most susceptible to the influence of a GP–GC relationship in forming age-related attitudes? Answers to these questions could inform the design of ageism-reduction interventions. Fourth, the current study focused on positive intergenerational contact, however, young people may also have unpleasant contact experiences with their grandparents, which might bring detrimental effects on attitudes toward older people. Future studies are needed to explore the joint, and probably asymmetry effects [62] of positive and negative GP-GC contact. Moreover, though including both explicit and implicit measures of attitudes toward older people is a merit of the current study, we acknowledge that Kogan’s Attitude toward Older People Scale is not the best measure of explicit ageism, given the ageist language included in the items and inconsistent evidence on convergent validity and criterion validity of the scale [63]. Measures with better psychometric properties, such as the Expectations Regarding Aging scale [64] should be used to assess explicit ageism in future studies.

Despite these limitations, we believe that our findings have important implications for reducing ageism. Our findings on the positive effects of contact with grandparents underscore the importance of promoting intergenerational contact within the family as a starting point to reduce prejudice toward older adults in age-segregated modern societies [4]. Institutional support is a known facilitator of positive intergroup contact [9]. Cultural events such as “Grandparents’ Day” could potentially enhance opportunities and quality of interactions between the younger generation and their grandparents. The current findings also shed light on how to extend the benefits of GP–GC contact outside the family. Positive views toward specific older adults (e.g., one’s grandparents) tend to generalize to the entire outgroup when older adults known intimately are perceived as typical representatives of all older people. By manipulating the grandchildren’s thoughts about their grandparents (e.g., by writing about ways in which grandparents are the same as other older adults), ageism reduction programs could increase the relationships between feelings for grandparents and more general attitudes toward older people.

### Electronic supplementary material

Below is the link to the electronic supplementary material.

Supplementary Material 1

## Data Availability

The datasets generated and/or analyzed during the current study are not publicly available due to institutional ethics regulations but are available from the corresponding author upon reasonable request.

## Footnotes

1 We also ran analyses with both contact indicators (quantity and quality), as well as their interaction terms with typicality in one model. The patterns of results were similar to those presented in Table 3, with the exception that the interaction between contact quality and typicality in predicting explicit attitudes did not reach a significant level at 0.05. This is probably due to the relatively small sample size and lack of statistical power of the current study.

2 Analyses with both predictors (i.e., contact quantity and contact quality) in the model simultaneously (carried out through path analysis) revealed a similar pattern of mediation effects of intergroup anxiety and IOS in the linkage between GP-GC contact and explicit attitudes. The only exception lies in the mediation effect of intergroup anxiety in the linkage between contact quality and attitudes toward older people, which did not reach a significant level at 0.05.

3 This pattern of findings was held in follow-up analyses with contact quantity also included in the model.

## Abbreviations

GP-GC: Grandparent and Grandchild
IOS: Inclusion of Other in the Self
KAOP: Kogan’s Attitude toward Older People Scale
SC-IAT: Single Category Implicit Association Test
BC CI: Bias-Corrected 95% Confidence Interval
IE: Indirect Effect

## References

[1] Population Division of the United Nations Department of Economic and Social Affairs (UN DESA). World population prospects 2022: Summary of results. 2022. https://www.un.org/development/desa/pd/content/World-Population-Prospects-2022.

[2] North MS, Fiske ST (2015). Modern attitudes toward older adults in the aging world: a cross-cultural meta-analysis. Psychol Bull.

[3] Luo B, Zhou K, Jin EJ, Newman A, Liang J (2013). Ageism among college students: a comparative study between U.S. and China. J Cross-Cult Gerontol.

[4] North MS, Fiske ST (2012). An inconvenienced youth? Ageism and its potential intergenerational roots. Psychol Bull.

[5] Swift HJ, Chasteen AL (2021). Ageism in the time of COVID-19. Group Processes and Intergroup Relations.

[6] Masse M, Meire P (2009). Facing age stigmatization: the impact on self-esteem and the role of protective strategies among older adults. J Nutr Health Aging.

[7] Abrams D, Eller A, Bryant J (2006). An age apart: the effects of intergenerational contact and stereotype threat on performance and intergroup bias. Psychol Aging.

[8] Levy BR (2009). Stereotype embodiment: a psychosocial approach to aging. Curr Dir Psychol Sci.

[9] Allport G (1954). The nature of prejudice. Garden City.

[10] Pettigrew TF (1998). Intergroup contact theory. Ann Rev Psychol.

[11] Li L, Li L, Wan Y (2021). Role conflict and adjustment of intergenerational raising and social participation of the elderly from the perspective of active aging. Adm Reform.

[12] Liou CL (2017). A comparative study of undergraduates’ attitudes toward aging in Taiwan and the United States through student drawings. Int J Aging Hum Dev.

[13] Newsham TM, Schuster AM, Guest MA, Nikzad-Terhune K, Rowles GD (2021). College students’ perceptions of old people compared to grandparents. Educ Gerontol.

[14] Zuo B, Wen F, Zhu X (2007). College students’ age stereotypes toward young and elderly people. Chin J Appl Psychol.

[15] Luan W, Wen X (2016). Ageism among undergraduates in aging China — taking Baoding city as an example. Social Work and Management.

[16] Zhang S, Liu YH, Zhang HF, Meng LN, Liu PX (2016). Determinants of undergraduate nursing students’ care willingness towards the elderly in China: attitudes, gratitude and knowledge. Nurse Educ Today.

[17] Wilson TD, Lindsey S, Schooler TY (2000). A model of dual attitudes. Psychol Rev.

[18] Cherry KE, Allen PD, Denver JY, Holland KR (2015). Contributions of social desirability to self-reported ageism. J Appl Gerontol.

[19] Chopik WJ, Giasson HL (2017). Age differences in explicit and implicit age attitudes across the life span. Gerontologist.

[20] Drury L, Hutchinson P, Abrams D (2016). Direct and extended intergenerational contact and young people’s attitudes towards older adults. Br J Psychol.

[21] Harwood J, Hewstone M, Paolini S, Voci A (2005). Grandparent-grandchild contact and attitudes toward older adults: moderator and mediator effects. Personal Soc Psychol Bull.

[22] Flamion A, Missotten P, Marquet M, Adam S (2019). Impact of contact with grandparents on children’s and adolescents’ views on the Elderly. Child Dev.

[23] Cai H, Huang Z, Lin L, Zhang M, Wang X, Zhu H (2020). The change of chinese psychology over the past half century: empirical research. Adv Psychol Sci.

[24] Richards Z, Hewstone M (2001). Subtyping and subgrouping: processes for the prevention and promotion of stereotype change. Personality and Social Psychology Review.

[25] Zhang YB, Paik S, Xing C, Harwood J (2018). Young adults’ contact experiences and attitudes toward aging: age salience and intergroup anxiety in South Korea. Asian J Communication.

[26] Cherry KE, Allen PD, Jackson EM, Hawley KS, Brigman S (2010). Knowledge of normal and pathological memory aging in college students, social workers, and health care professionals. Educ Gerontol.

[27] Bousfield C, Hutchison P (2010). Contact, anxiety, and Young People’s attitudes and behavioral intentions towards the Elderly. Educ Gerontol.

[28] Prestwich A, Kenworthy JB, Wilson M, Kwan-Tat N (2008). Differential relations between two types of contact and implicit and explicit racial attitudes. Br J Soc Psychol.

[29] Tam T, Hewstone M, Harwood J, Voci A, Kenworthy J (2006). Intergroup contact and grandparent-grandchild communication: the effects of self-disclosure on implicit and explicit biases against older people. Group Processes and Intergroup Relations.

[30] Vezzali L, Giovannini D (2011). Intergroup contact and reduction of explicit and implicit prejudice towards immigrants: a study with italian businessmen owning small and medium enterprises. Qual Quantity.

[31] Binder J, Zagefka H, Brown R, Funke F, Kessler T, Mummendey A (2009). Does contact reduce prejudice or does prejudice reduce contact? A longitudinal test of the contact hypothesis among majority and minority groups in three european countries. J Personal Soc Psychol.

[32] Brown R, Hewstone H, Zanna MP (2005). An integrative theory of intergroup contact. Advances in experimental social psychology.

[33] Dovidio JF, Love A, Schellhaas FM, Hewstone M (2017). Reducing intergroup bias through intergroup contact: twenty years of progress and future directions. Group Processes & Intergroup Relations.

[34] Stephan WG, Stephan CW (1985). Intergroup anxiety. Journal of social issues. Stereotyping and prejudice against older persons.

[35] Aron A, Aron EN, Tudor M, Nelson G (1991). Close relationships as including other in self. J Personal Soc Psychol.

[36] Page-Gould E, Mendoza-Denton R, Alegre JM, Siy JO (2010). Understanding the impact of cross-group friendship on interactions with novel outgroup members. J Personal Soc Psychol.

[37] Aron A, McLaughlin-Volpe T, Mashek D, Lewandowski G, Wright SC, Aron EN (2004). Including others in the self. Eur Rev Social Psychol.

[38] Wright SC, Aron A, McLaughlin-Volpe T, Ropp SA (1997). The extended contact effect: knowledge of cross–group friendships and prejudice. J Personal Soc Psychol.

[39] Turner RN, Hewstone M, Voci A, Vonofakou C (2008). A test of the extended intergroup contact hypothesis: the mediating role of intergroup anxiety, perceived ingroup and outgroup norms, and inclusion of the outgroup in the self. J Personal Soc Psychol.

[40] Du P, Li L. Long-term trends projection of China’s population aging in the new era. J Renmin Univ China. 2021;196–109. 10.3969/j.issn.1000-5420.2021.01.010.

[41] Aron A, Aron EN, Smollan D (1992). Inclusion of other in the self-scale and the structure of interpersonal closeness. J Personal Soc Psychol.

[42] Liu Y, Yang W, Ma L, While A, Ye W, Chen J (2014). Reliability and validity of the chinese version of Kogan’s attitudes towards old people scale. Chin J Nurs.

[43] Karpinski A, Steinman RB (2006). The single Category Implicit Association Test as a measure of Implicit Social Cognition. J Personal Soc Psychol.

[44] Levy BR, Langer E (1994). Aging free from negative stereotypes: successful memory in China among the american deaf. J Psychol Social Psychol.

[45] Officer A, Thiyagarajan JA, Schneiders ML, Nash P (2020). Ageism, healthy life expectancy and population ageing: how are they related?. Int J Environ Res Public Health.

[46] Lasher KP, Faulkender PJ (1993). Measurement of aging anxiety: development of the anxiety about aging scale. Int J Aging Hum Dev.

[47] Donizzetti AR. Ageism in an aging society: the role of knowledge, anxiety about aging, and stereotypes in young people and adults. International Journal of Environmental Research and Public Health. 2019; 16(9), 1329–1340. 10.3390/ijerph16081329. 10.3390/ijerph16081329 PMC6517915 31013873

[48] Cooney C, Minahan J, Siedlecki KL (2021). Do feelings and knowledge about aging predict ageism?. J Appl Gerontol.

[49] Greenberg J, Schimel J, Martens A, Ageism, Nelson TD (2002). Denying the face of the future. Ageism: stereotyping and prejudice against older persons.

[50] Aiken LS, West SG (1991). Multiple regression: testing and interpreting interactions.

[51] Preacher KJ, Hayes AF (2008). Asymptotic and resampling strategies for assessing and comparing indirect effects in multiple mediator models. Behav Res Methods.

[52] Hayes AF (2013). Introduction to mediation, moderation, and conditional process analysis: a regression-based approach.

[53] Muller D, Judd CM, Yzerbyt VY (2005). When moderation is mediated and mediation is moderated. J Personal Soc Psychol.

[54] Preacher KJ, Rucker DD, Hayes AF (2007). Assessing moderated mediation hypotheses: theory, methods, and prescriptions. Multivar Behav Res.

[55] Bratt C, Abrams D, Swift HJ (2020). Supporting the old but neglecting the young? The two faces of ageism. Dev Psychol.

[56] Lin Y, Peng C (2020). College students’ attitudes toward older adults. Community Psychol Res.

[57] Fazio RH, Olson MA (2003). Implicit measures in social cognition research: their meaning and use. Ann Rev Psychol.

[58] Voci A, Hewstone M (2003). Intergroup contact and prejudice toward immigrants in Italy: the mediational role of anxiety and the moderational role of group salience. Group Processes & Intergroup Relations.

[59] Pettigrew TF, Tropp LR (2008). How does intergroup contact reduce prejudice? Meta-analytic tests of three mediators. Eur J Social Psychol.

[60] Turner RN, Hewstone M, Voci A (2007). Reducing explicit and implicit outgroup prejudice via direct and extended contact: the mediating role of self-disclosure and intergroup anxiety. J Personal Soc Psychol.

[61] Vezzali L, Lolliot S, Trifiletti E, Cocco VM, Rae JR, Capozza D, Hewstone M (2023). Effects of intergroup contact on explicit and implicit outgroup attitudes: a longitudinal field study with majority and minority group members. Br J Soc Psychol.

[62] Fuochi G, Voci A, Boin J, Hewstone M (2020). Close to me: the importance of closeness versus superficiality in explaining the positive-negative contact asymmetry. Eur J Social Psychol.

[63] Ayalon L, Dolberg P, Mikulionienė S, Perek-Białas J, Rapolienė G, Stypinska J (2019). A systematic review of existing ageism scales. Ageing Res Rev.

[64] Sarkisian CA, Neil SW, Hays RD, Mangione CM (2005). Development of the 12-item expectations regarding aging survey. Gerontologist.

