# Supplementary material for: Contact with grandparents and young people’s explicit and implicit attitudes toward older adults
Source: BMC Psychol. 2023 Sep 26;11:289. doi: 10.1186/s40359-023-01344-7 (PMC10521500; doi:10.1186/s40359-023-01344-7)
Supplement: Supplementary file 1 — Supplementary Material 1 [file 40359_2023_1344_MOESM1_ESM.docx]

Appendix

Target Concepts and Attributive Words Used in the SC-IAT

| Target words representing older people |  | Attributive words | |
| --- | --- | --- | --- |
|  |  | Positive | Negative |
| retirement |  | experienced | slow-moving |
| walking stick |  | active | feeble |
| gray hair |  | frugal | sick |
| wrinkle |  | kind | old-fashioned |
| nursing home |  | loving | nagging |
| public square dancing |  | mellow | forgetful |
| sunset |  | optimistic | dependent |
| health protection medicine |  | wise | gullible |
